# Enhanced Visualization of the Distal Myotomy Endpoint during Peroral Endoscopic Myotomy Using Indocyanine Green With Infrared Imaging

**DOI:** 10.1002/deo2.70250

**Published:** 2025-12-10

**Authors:** Yuichiro Ikebuchi, Takuki Sakaguchi, Moe Eizawa, Tsuyoshi Mikamo, Kazuhiro Takemoto, Yuki Fujii, Ryohei Ogihara, Yu Kamitani, Mirai Edano, Hidehito Kinoshita, Hiroki Kurumi, Takashi Hasegawa, Koichiro Kawaguchi, Kazuo Yashima, Hajime Isomoto

**Affiliations:** ^1^ Department of Multidisciplinary Internal Medicine Division of Gastroenterology and Nephrology Tottori University Hospital Tottori Japan; ^2^ Division of Internal Medicine Motomachi Hospital Tottori Japan

**Keywords:** esophageal achalasia, esophageal motility disorders, esophagus, indocyanine green, myotomy

## Abstract

**Background:**

Peroral endoscopic myotomy (POEM) is an effective treatment for achalasia; however, accurately identifying the distal extent of myotomy remains a technical challenge. Indocyanine green with infrared imaging (ICG‐IRI) may facilitate the intraoperative visualization of the esophagogastric junction.

**Methods:**

We evaluated 39 patients who underwent POEM using ICG‐IRI. The primary outcome was the success rate of ICG‐IRI, defined as a clear endoscopic visualization of fluorescence at the distal myotomy endpoint. The secondary outcomes included technical success, changes in integrated relaxation pressure, and Eckardt scores before and after treatment.

**Results:**

The technical success rate was 100%, and ICG‐IRI was successful in 94.9% of the cases (37/39). The median integrated relaxation pressure decreased from 26.9 to 10.8 mmHg, and the median Eckardt score improved from 5.0 to 1.0 (both *p* < 0.01). No adverse events were attributed to the ICG‐IRI.

**Conclusions:**

The ICG‐IRI method is a reliable visual aid for identifying distal myotomy endpoints during POEM. This technique may enhance the procedural accuracy and improve clinical outcomes.

## Graphical abstract

Indocyanine green with infrared imaging (ICG‐IRI) provides real‐time fluorescence visualization of the distal myotomy endpoint during peroral endoscopic myotomy (POEM). In this study of 39 patients, ICG‐IRI achieved a 94.9% success rate in fluorescence detection and significantly improved esophageal relaxation and symptoms. This simple and safe technique enhances procedural precision and may reduce incomplete myotomy during POEM.

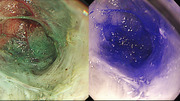

**Abbreviations**
EGJ: esophagogastric junction
HRM: high‐resolution manometry
ICG‐IRI: indocyanine green with infrared imaging
IRP: integrated relaxation pressure
LES: lower esophageal sphincter
POEM: peroral endoscopic myotomy.

## 1 Introduction

Achalasia is a rare primary esophageal motility disorder characterized by the absence of esophageal peristalsis and failure of the lower esophageal sphincter (LES) to relax during swallowing [1]. The etiology remains unclear, but autoimmune‐mediated inflammation and degeneration of the myenteric plexus, possibly triggered by viral infections, such as herpes simplex virus, have been suggested [2]. This dysfunction causes progressive dysphagia, regurgitation, and weight loss due to functional obstruction at the esophagogastric junction (EGJ).

The management of achalasia aims to relieve distal esophageal outflow obstruction, as no therapy reverses the underlying neuropathy. Before the introduction of peroral endoscopic myotomy (POEM), standard treatment included pneumatic dilation or endoscopic botulinum toxin injection, with laparoscopic Heller myotomy reserved for refractory cases. POEM, introduced by Inoue et al. in 2010 [3], has become a widely adopted, minimally invasive, and definitive therapy, providing symptomatic relief rates comparable to or better than Heller myotomy [4, 5]. Thus, POEM is now established as a first‐line treatment for achalasia.

The technical success of POEM depends on adequate division of the LES while minimizing complications. Achieving a sufficient myotomy length, particularly its distal extension into the gastric cardia, is critical because incomplete LES division is a major cause of persistent dysphagia ​[6]. However, extending the myotomy too far beyond the EGJ increases the risk of mucosal perforation or bleeding and may worsen postoperative reflux [7]. Therefore, precise identification of the EGJ is essential. Endoscopists typically confirm gastric extension by anatomical landmarks, scope insertion depth, and tactile resistance [7], but locating the EGJ within the submucosal tunnel remains difficult.

To address this, several adjunctive techniques have been developed. The double‐scope POEM method introduces a second endoscope into the stomach to visualize the EGJ from below and provide transillumination feedback [8]. The development and adoption of such techniques highlight the clinical importance of accurate EGJ identification and support the use of enhanced visualization to improve procedural completeness.

Despite the benefits of the double‐scope technique, it requires additional equipment and coordination and is not universally available. This has spurred interest in alternative methods to assist POEM operators in marking the intended myotomy endpoint. One novel approach involves the use of indocyanine green (ICG) dye with infrared imaging (ICG‐IRI) to highlight the target site. ICG is a fluorescent contrast agent with a long record of safety in various surgical fields. It strongly absorbs near‐infrared light around 780–805 nm and emits fluorescence at slightly longer wavelengths (approximately 810–830 nm). Infrared imaging (IRI) captures both reflected and fluorescent signals in real time, allowing visualization of ICG distribution within tissue. In endoscopy, ICG can be injected into the submucosa at or just distal to the EGJ, and switching to infrared mode enables clear visualization of the fluorescent signal that delineates the LES area.

We recently demonstrated the feasibility of this technique for POEM [9]. In our study, intratunnel submucosal ICG injection combined with infrared visualization provided a bright fluorescence landmark that marked the distal end of the submucosal tunnel. This guidance enabled the endoscopist to confidently extend the myotomy to a predefined endpoint, helping prevent both incomplete division and inadvertent overextension on the gastric side​. Given the importance of accurate endpoint marking for a successful myotomy, ICG‐IRI may represent a significant advancement in the endoscopic management of achalasia. However, data on its clinical utility in POEM are limited to preliminary reports.

Therefore, in this study, we aimed to evaluate the efficacy and safety of ICG‐IRI in patients undergoing POEM for achalasia. Specifically, we assessed whether fluorescence‐guided imaging facilitates a more precise identification of the distal myotomy endpoint, thereby improving the completeness of myotomy and potentially enhancing patient outcomes.

## 2 Methods

### 2.1 Patients

This study included patients who underwent POEM at the Tottori University Hospital between July 2020 and December 2024. Because the use of ICG in POEM is not formally approved in Japan, written informed consent was obtained from all patients prior to the procedure in accordance with institutional ethical guidelines. Patients aged 20 years or older who were diagnosed with esophageal achalasia or achalasia‐related motility disorders and underwent POEM were included. Patients were excluded if they had a known ICG allergy or did not provide consent. This study was approved by the Ethics Committee of Tottori University Hospital (approval number: 20A176) and was conducted following the institutional guidelines and the Declaration of Helsinki.

### 2.2 Database Structure and Variables

The collected variables included age at the time of high‐resolution manometry (HRM), sex, body mass index, prior treatment, Eckardt score, HRM‐based diagnosis, and integrated relaxation pressure (IRP) values. Symptom severity was assessed using the Eckardt score, with higher scores (maximum 12) indicating greater severity [10].

All patients underwent barium esophagography prior to POEM to assist in the diagnosis and morphological classification of achalasia. Based on esophagography findings, achalasia was classified into straight, sigmoid, and advanced sigmoid types. The sigmoid type was defined by an esophageal flexion angle (α) <135° but ≥90°, whereas the advanced sigmoid type was defined by an angle <90° [11]. HRM‐based diagnoses were determined according to the Chicago Classification, version 4.0 [12].

To assess the deglutitive relaxation of the LES, the IRP was calculated as the lowest cumulative pressure over 1‐ and 4‐second intervals within a 10‐second post‐swallow window, as recorded by the e‐sleeve sensor across the EGJ. For interdevice comparability, the IRP values were converted using the Starlet system (Star Medical Inc., Tokyo, Japan) as previously described. An IRP ≥26 mmHg was considered elevated, indicating incomplete LES relaxation [13].

The procedural characteristics and clinical outcomes of the patients who underwent POEM were evaluated. Adverse events were graded using the Clavien‐Dindo classification system (grades I–V) [14], with events of grade II or higher considered adverse. Technical success was defined as the successful completion of POEM. The ICG‐IRI method was considered successful when the ICG fluorescence was clearly visualized endoscopically. The Eckardt score and HRM were obtained approximately 2–3 months after the procedure, and EGD for reflux evaluation was also scheduled during the same period. Reflux esophagitis was graded as A–D according to the Los Angeles classification [15].

### 2.3 ICG‐IRI Technique

**FIGURE 1:**
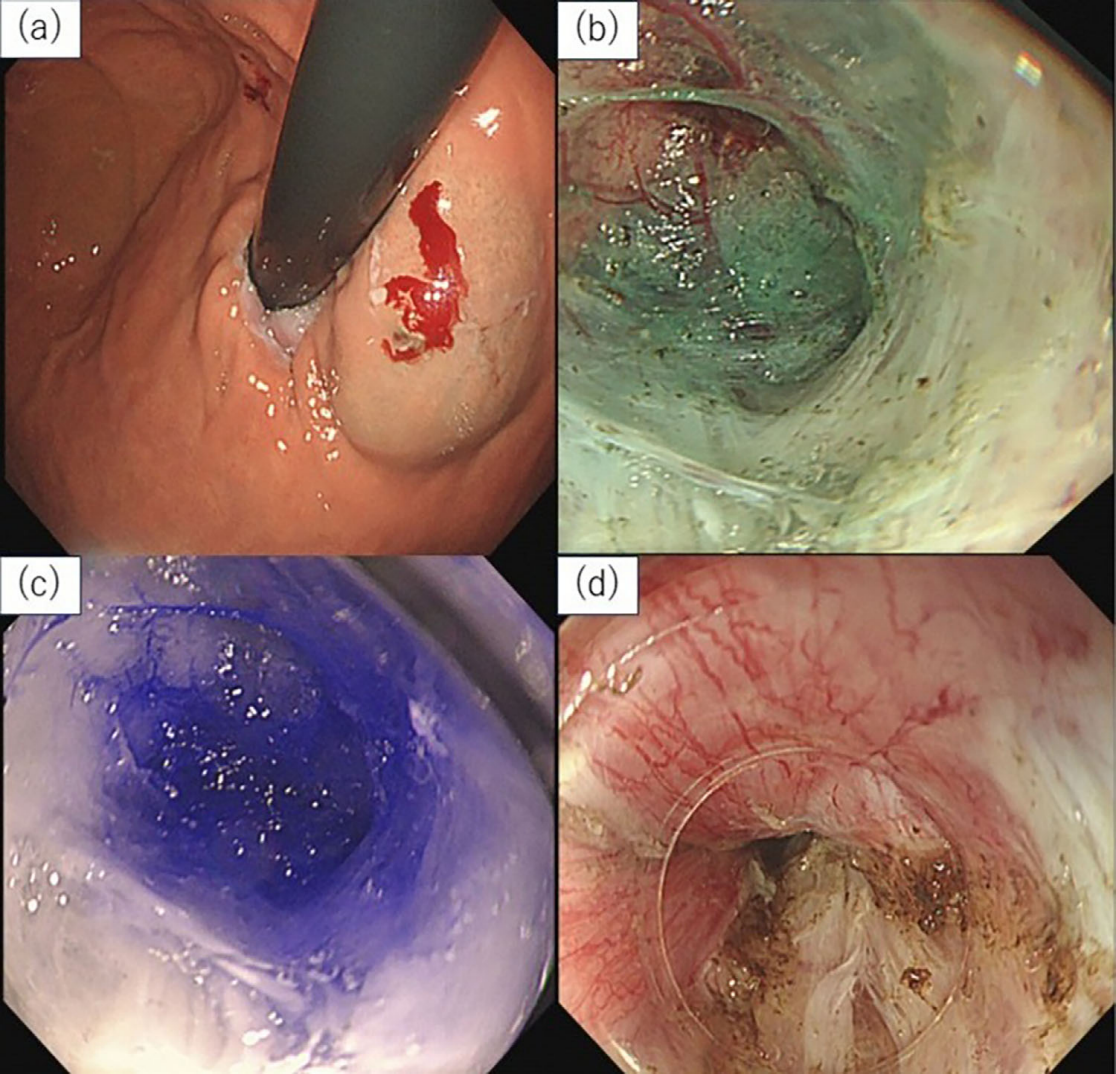
POEM procedure using the ICG‐IRI method. (a) Indocyanine green was injected into the submucosal layer to mark the distal myotomy endpoint before POEM. (b) A conventional POEM was performed, and white light observation confirmed the arrival at the gastric side. (c) This image shows the same location as in (b), observed using near‐infrared imaging. Near‐infrared imaging of the same site using ICG‐IRI reveals clear visualization of the marked area. (d) After confirmation with ICG‐IRI, myotomy was performed using the standard POEM technique. ICG‐IRI, indocyanine green with infrared imaging; POEM, peroral endoscopic myotomy.

ICG (Daiichi Sankyo Co., Ltd., Tokyo, Japan) was used for POEM via ICG‐IRI (Video S1). A small amount of normal saline was injected approximately 1–2 cm distal to the EGJ on the gastric lesser curvature to create a submucosal cushion, and then 0.5 mL of 5 mg/mL ICG was injected. The injection site was set at 1–2 cm distal to the EGJ based on the report by Shiwaku et al., which indicated that excessive extension of the myotomy onto the gastric side increases the risk of postoperative reflux esophagitis [16]. For patients with diffuse esophageal spasm (DES) and hypercontractile esophagus (HE), however, ICG was injected into the esophageal mucosa slightly proximal to the EGJ. This target site was chosen to align with the optimal myotomy endpoint as recommended in current POEM guidelines and previous reports [17, 18]. The injection site was standardized using the estimated distance from the squamocolumnar junction and scope insertion depth (Figure 1a). POEM was then performed using the standard technique: submucosal injection of saline mixed with indigo carmine, followed by mucosal incision and creation of a submucosal tunnel, all via the posterior (5–6 o'clock) approach. The anticipated tunnel extension on the gastric side was estimated based on the endoscope insertion depth. Once this position was reached (Figure 1b), enhanced visualization of the ICG signal as a blue pseudo‐color was confirmed using an endoscopic imaging system (EVIS LUCERA ELITE RQ260Z; Olympus Corporation, Tokyo, Japan) equipped with an IRI capability (Figure 1c). If no fluorescence was observed, the tunnel was extended until the signal was visible. Confirmation of fluorescence was defined as a well‐defined signal identified by the operator at the predetermined distal endpoint during the procedure. Once the distal extent was confirmed, a myotomy was performed (Figure 1d), and the mucosal entry site was closed using endoscopic clips.

### 2.4 Outcomes

The primary outcome was the success rate of the ICG‐IRI method, defined as the endoscopic visualization of fluorescence at the distal myotomy endpoint. Secondary outcomes included technical success of the POEM procedure, changes in IRP and Eckardt scores before and after POEM, and the presence of postoperative reflux esophagitis as classified using the Los Angeles classification, the use of proton pump inhibitor (PPI) or potassium‐competitive acid blocker (PCAB), and the proportion of open‐type gastric atrophy. Paired comparisons of pre‐ and post‐treatment IRP and Eckardt scores were performed using the Wilcoxon signed‐rank test. Statistical significance was set at p <0.05. significant.

### 2.5 Statistical Analysis

Continuous variables, including Eckardt scores, IRP, and procedure time, are reported as medians with interquartile ranges (IQRs). The Wilcoxon signed‐rank test was used to compare paired pre‐ and post‐treatment values of the Eckardt score and IRP. Categorical outcomes such as the presence of gastroesophageal reflux disease and successful ICG‐IRI visualization are summarized as frequencies and percentages. Statistical significance was set at a two‐tailed *p*‐value <0.05. All analyses were performed using Python (version 3.11) and the SciPy statistical library.

## 3 Results

Thirty‐nine patients (11 females, 28.2%; median age, 63 [IQR, 48–74] years) underwent POEM assisted by the ICG‐IRI method (Table 1). The median body mass index was 22.4 kg/m^2^ (IQR: 20.1–25.7). Among these, open‐type gastric atrophy was observed in 28.2% (11/39), including eight cases after Helicobacter pylori eradication and three with unknown H. pylori status. In addition, 35.9% (14/39) had been on PPI or PCAB before POEM. According to the Chicago Classification, type I achalasia was the most common subtype (41.0%), followed by type II achalasia (33.3%), and other subtypes, including type III, DES, and HE. Morphologically, the straight‐type achalasia was predominant (79.5%).

**TABLE 1:** Baseline clinical and endoscopic characteristics of patients who underwent indocyanine green with infrared imaging (ICG‐IRI)‐assisted peroral endoscopic myotomy (POEM).

	ICG‐IRI (*n* = 39)
Age, years, median (IQR)	63 (48–74)
Female sex, *n* (%)	11 (28.2)
BMI, kg/m^2^, median (IQR)	22.4 (20.1–25.7)
Subtype of HRM diagnosis based on Chicago Classification, *n* (%):	
Type I	16 (41.0)
Type II	13 (33.3)
Type III	2 (5.1)
DES	2 (5.1)
HE	6 (15.3)
Achalasia type, *n* (%):	
Straight type	31 (79.5)
Sigmoid type	6 (15.3)
Advanced sigmoid type	2 (5.1)
Pre‐treatment: Balloon dilation, *n* (%)	8 (20.5)
Open‐type gastric atrophy, *n* (%)	11 (28.2)
After H. pylori eradication	8 (20.5)
Unknown H. pylori status	3 (7.7)
Regular use of PPI or PCAB before POEM, *n* (%)	14 (35.9)
Pre‐POEM IRP, mmHg, median (IQR)	26.9 (17.1–39.6)
Pre‐POEM Eckardt score, median (IQR)	5 (3.5–7)

Abbreviations: BMI, body mass index; DES, diffuse esophageal spasm; HE, hypercontractile esophagus; HRM, high‐resolution manometry; ICG‐IRI, indocyanine green with infrared imaging; IQR, interquartile range; IRP, integrated relaxation pressure; POEM, peroral endoscopic myotomy.

All 39 patients successfully underwent POEM, with a technical success rate of 100% (Table 2). The ICG‐IRI method was successful in 94.9% of cases (*n* = 37), with clear endoscopic visualization of fluorescence signals, enabling precise identification of the myotomy endpoint. The fluorescence was clearly identified on the gastric side without proximal (esophageal) diffusion, confirming accurate submucosal localization. In the two cases where ICG‐IRI failed, the distal tunnel extent was confirmed using a double‐scope technique as per standard POEM. The median procedure time was 61 min (IQR: 50–79 min). The median myotomy length was 6.0 cm (IQR: 4.5–8.0) in the esophagus and 2.0 cm (IQR: 1.0–2.0) on the gastric side.

**TABLE 2:** Procedural and post‐treatment outcomes after indocyanine green with infrared imaging (ICG‐IRI)‐assisted peroral endoscopic myotomy (POEM).

	ICG‐IRI (*n* = 39)
Procedure time, min, median (IQR)	61 (50–79)
Myotomy length in the esophagus, cm, median (IQR)	6 (4.5–8.0)
Myotomy length in the stomach, cm, median (IQR)	2 (1.0–2.0)
Adverse events, *n* (%)	0 (0)
Technical success, *n* (%)	39 (100)
ICG‐IRI success, *n* (%)	37 (94.9)
Symptomatic GERD, *n* (%)	2 (5.1)
Reflux esophagitis:	
LA classification grade C or higher, *n* (%)	2 (5.1)
Post‐POEM IRP, mmHg, median (IQR)	10.8 (7.9–15.0)
Post‐POEM Eckardt score, median (IQR)	1 (0–1)

Abbreviations: ICG‐IRI, indocyanine green with infrared imaging; IQR, interquartile range; IRP, integrated relaxation pressure; LA, Los Angeles classification; POEM, peroral endoscopic myotomy.

This treatment resulted in significant clinical improvement. The median Eckardt score decreased from 5.0 (IQR: 3.5–7.0) preoperatively to 1.0 (IQR: 0–1.0) postoperatively (*p* = 0.016). Similarly, the median IRP declined from 26.9 mmHg (IQR: 17.1–39.6) to 10.8 mmHg (IQR: 7.9–15.0) after treatment (*p* = 0.016), indicating effective myotomy.

Symptomatic GERD occurred in 5.1% (2/39) after POEM. Reflux esophagitis of Los Angeles grade C was observed in 5.1% (2/39) of patients, but none of them reported reflux symptoms. No adverse events related to ICG or IRI were observed.

## 4 Discussion

This study demonstrated that ICG‐IRI is a feasible and highly effective adjunct for guiding POEM, with a 94.9% success rate in fluorescence visualization at the distal myotomy endpoint. This real‐time visual cue likely contributed to the consistent completeness of myotomy, as evidenced by significant posttreatment improvements in Eckardt scores and IRP. By enabling the precise identification of the EGJ within the submucosal tunnel, the ICG‐IRI method addresses a key technical challenge in POEM and targets a common point of procedural failure. When treatment success was defined as an Eckardt score of ≤3, all patients in this study achieved clinical success, which is comparable to short‐term outcomes (92.9%–94.9%) reported in previous systematic reviews and meta‐analyses [19, 20, 21, 22].

Several methods have been proposed to ensure the accurate identification of the distal myotomy endpoint during POEM, each with distinct advantages and limitations. The double‐scope technique allows retroflexed visualization of the EGJ and was shown by Grimes et al. to increase gastric myotomy length and reduce the incidence of underdissection [8]. Recently, fluorescence‐based techniques and anatomical landmark‐guided strategies have attracted considerable attention. Minami et al. reported the utility of ICG injection into the gastric cardia to enhance EGJ localization during submucosal tunneling [23]. Tanaka et al. subsequently demonstrated that using two penetrating vessels as anatomical landmarks to guide the distal extent of posterior POEM preserves oblique (sling) fibers and significantly reduces the incidence of post‐POEM gastroesophageal reflux [24]. Uchima et al. further validated this approach in a Western cohort, supporting the use of penetrating vessels as a practical and reproducible guide for determining the appropriate myotomy endpoint [25].

Our results suggest that the ICG‐IRI method is a promising alternative that may overcome the limitations of existing techniques. In a previous pilot study, we demonstrated that the submucosal injection of ICG into the LES, visualized via near‐IRI, produced a bright, easily recognizable fluorescent marker at the distal end of the tunnel. In contrast to submucosal ICG injection without IRI, which yields only faint discoloration, ICG‐IRI provides a markedly clearer, high‐contrast signal. This real‐time visual feedback facilitates precise dissection even when traditional anatomical landmarks are obscured.

Conceptually, ICG‐IRI offers benefits similar to double‐scope POEM in confirming adequate gastric extension, but requires only a single endoscope. Near‐infrared capability is integrated into the imaging system of the primary endoscope, eliminating the need for additional instruments or personnel. In our series, the median procedure time was approximately 61 min, which is consistent with the standard POEM duration [7]. This indicates that the additional steps of ICG injection and infrared mode activation had minimal impact on the workflow and did not significantly prolong the procedure.

On the other hand, ICG‐IRI failed in two cases. Upon reviewing the recorded videos, the main cause was considered to be inadvertent intramuscular injection, which led to attenuated submucosal fluorescence. Both cases were safely recovered by using double‐scope confirmation. These findings underscore the importance of ensuring accurate submucosal injection for the successful implementation of the ICG‐IRI method.

Notably, no adverse events were reported associated with ICG or IRI, confirming the safety of the technique. ICG has an established safety record in clinical practice [26, 27], and unlike dyes such as methylene blue, it does not permanently stain tissues or obscure the field. This favorable profile was confirmed in our study; no patient experienced allergic reactions or injection‐site complications, and incidental ICG spillage was inconsequential.

This study had several limitations. First, this was a single‐center, non‐randomized, observational study without a control group, which limits direct comparisons with conventional POEM or other adjunctive techniques. Although this study did not perform direct comparisons with the double‐scope or penetrating vessel methods, our findings suggest that ICG‐IRI may serve as a feasible alternative. To confirm these preliminary results, prospective randomized trials comparing these modalities—including endoscopic landmarks and double‐scope confirmation—should be conducted across multiple centers. Second, although the sample size was larger than that in prior pilot reports, it remained relatively small. Third, the ICG‐IRI method failed in two cases, suggesting that it may not be universally reliable, particularly in patients with anatomically altered or fibrotic submucosa. Finally, the requirement for specialized endoscopic equipment with near‐IRI capabilities may limit its broader adoption. The EVIS LUCERA ELITE RQ260Z system used in this study, which is equipped with IRI capability, is no longer commercially available and currently difficult to obtain. We hope that future‐generation endoscopy systems will incorporate similar or improved IRI functionality to facilitate broader clinical application.

In conclusion, ICG‐IRI is a feasible and safe technique that enables clear visualization of the distal myotomy endpoint. This adjunct may enhance the procedural precision and reduce the risk of incomplete myotomy. Although our findings are preliminary, they support the potential role of ICG‐IRI in standard POEM. Larger controlled studies are required to confirm its clinical impact and broader applicability.

## Author Contributions

Yuichiro Ikebuchi, Takuki Sakaguchi, Hidehito Kinoshita, and Hajime Isomoto contributed to the original study concept and design. Yuichiro Ikebuchi, Takuki Sakaguchi, Moe Eizawa, Tsuyoshi Mikamo, Kazuhiro Takemoto, Yuki Fujii, Ryohei Ogihara, Yu Kamitani, Mirai Edano, Hidehito Kinoshita, Hiroki Kurumi, Takashi Hasegawa, Koichiro Kawaguchi, Kazuo Yashima, and Hajime Isomoto were involved in data collection and sample management. Yuichiro Ikebuchi, Takuki Sakaguchi, Hidehito Kinoshita, and Hajime Isomoto analyzed and interpreted the data. All the authors critically revised the manuscript and approved the final version for publication.

## Conflicts of Interest

The authors declare no conflicts of interest.

## Funding

This study received no external funding.

## Ethics Statement

This study was approved by the Ethics Committee of Tottori University Hospital (approval number: 20A176) and was conducted in accordance with the institutional guidelines and the Declaration of Helsinki.

## Consent

Written informed consent was obtained from all patients prior to the procedure.

## Clinical Trial Registration

N/A

## Supporting information




**VIDEO S1** Video Demonstration of the POEM Procedure Using Indocyanine Green–Infrared Imaging (ICG‐IRI) Method.
